# How to successfully administer palliative treatment with a stent for malignant gastric outlet obstruction?

**DOI:** 10.3389/fmed.2022.967740

**Published:** 2022-08-09

**Authors:** Iruru Maetani

**Affiliations:** Division of Gastroenterology and Hepatology, Department of Internal Medicine, Toho University Ohashi Medical Center, Tokyo, Japan

**Keywords:** gastric outlet obstruction (GOO), gastroduodenal obstruction, palliation, self-expandable metal stent (SEMS), gastroenterostomy

## Abstract

Although endoscopic stenting (ES) has been widely used as a less-invasive palliation method for malignant gastric outlet obstruction (GOO), recent reports have highlighted issues related to the procedure. For successful treatment, various aspects must be assessed before considering the practices. First, it is necessary to eliminate cases with contraindications such as coexistence of distal small-bowel obstruction or perforation. Other factors potentially related to clinical failure (i.e., peritoneal carcinomatosis) may require consideration but remain controversial. ES has better short-term outcomes than surgical gastrojejunostomy (GJ). GJ has recently been considered preferable in cases with longer life expectancy because of superior sustainability. Various types of stents are now commercially available, but their ideal structure and mechanical properties have not yet been clarified. Covered metal stent may reduce stent obstruction but is prone to increase stent migration, and its significance remains uncertain. Subsequent chemotherapy after stenting should be considered, as it is expected to prolong patient survival without increasing the risk of adverse events. Furthermore, it may be helpful in preventing tumor ingrowth. In cases with GOO combined with biliary obstruction, biliary intervention is often difficult. Recently, endoscopic ultrasound-guided biliary drainage (EUS-BD) has been widely used as an alternative procedure for endoscopic transpapillary biliary drainage (ETBD). Despite the lack of consensus as to whether ETBD or EUS-BD is preferred, EUS-BD is useful as a salvage technique for cases where ETBD is difficult. To perform stent placement successfully, it is important to pay attention to the above points; however, many remaining issues need to be clarified in the future.

## Introduction

Malignant gastric outlet obstruction (GOO) is caused by highly advanced cancers such as gastric, pancreatic, and biliary cancers. As these are usually unresectable, a patient's quality of life is likely to deteriorate owing to obstructive symptoms and inability to consume orally. Endoscopic stenting (ES) with a self-expandable metal stent (SEMS) has emerged as an alternative to conventional gastrojejunostomy (GJ). The procedure was first performed in the early 1990s (1–3); since then, it has been widely used because it is less invasive and has a rapid effect. However, recent reports (4, 5) have highlighted the issues associated with this procedure. For successful treatment, it is essential to be aware of stent-related problems and the risk factors for various associated issues before considering stent placement. This review focuses on a variety of procedural perceptions to achieve successful treatment, including appropriate patient selection, advance preparation, precautions, and counter-measures for complicated pathological conditions.

## Considerations for endoscopic stenting

### General aspects

To achieve success, it is essential to eliminate the contraindications for the procedure, such as perforation, multiple luminal obstructions (particularly, the coexistence of distal small-bowel obstruction) (6, 7), and severely impaired gastric motility (7). Perforation related to GOO is quite rare, but we should be aware of possible intestinal perforation by a migrated preexisting biliary stent (8).

**Figure 1:**
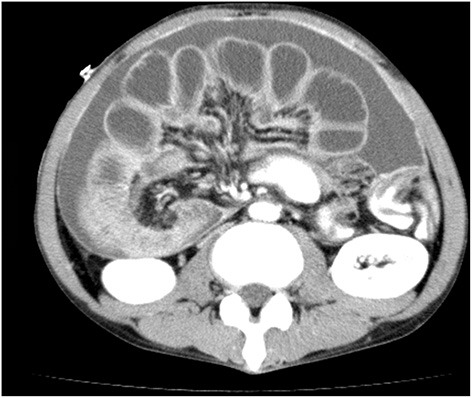
CT findings of cases with highly advanced gastric cancer. Multiple dilations and wall-thickening of the small bowel suggest multiple small-bowel obstructions are found.

**Figure 2:**
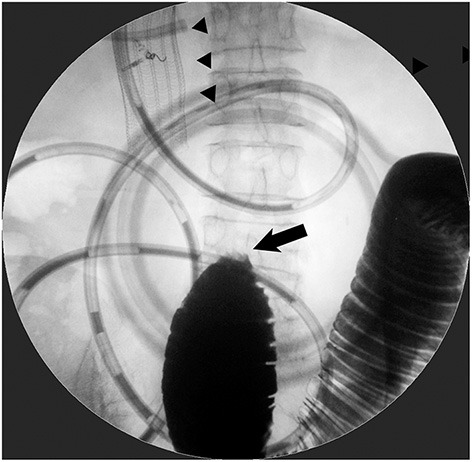
Patient with the coexistence of distal small-bowel obstruction. Stent placement for D2 obstruction from gallbladder cancer did not improve obstructive symptoms at all. Contrast examination from a decompression catheter through duodenal SEMS (arrowhead) depicted a complete jejunal obstruction (arrow), which required subsequent surgical jejuno-jejunostomy.

**Figure 3:**
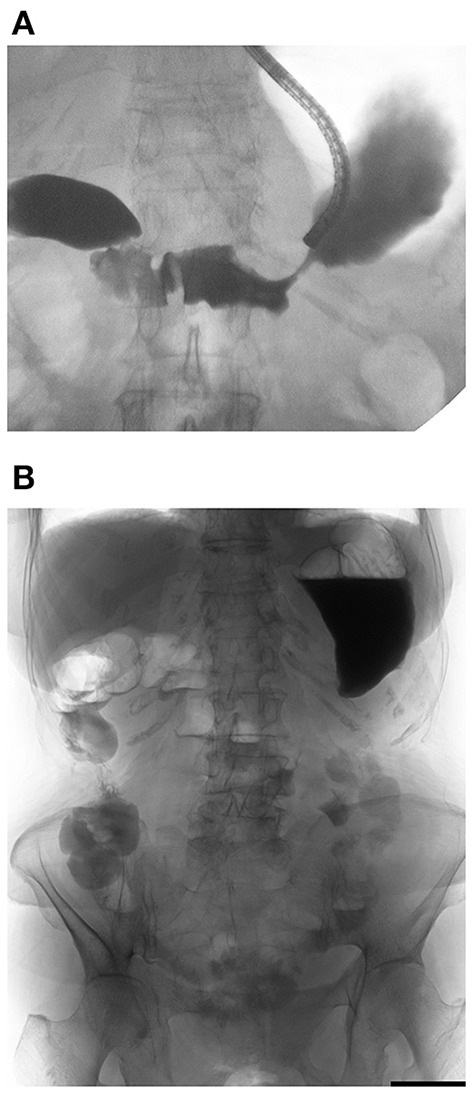
Water-soluble contrast challenge. **(A)** Water-soluble contrast was injected beyond the obstruction. **(B)** Plain X-ray taken 4 h after injection showed contrast reached the large intestine, which means well small bowel transit without the coexistence of small-bowel obstruction.

Restoration of the gastrointestinal tract continuity, which is the main goal of the procedure, cannot be achieved in patients with coexisting distal small-bowel obstruction. Before considering ES, it is necessary to clarify whether distal small bowel obstruction is present or not (9). Multiple small-bowel obstructions due to peritoneal dissemination can be detected on computed tomography (CT) (Figure 1); however, a solitary distal obstruction may not be easily detected, as GOO symptoms can mask those of distal small bowel obstruction (Figure 2). In such cases, administration of a water-soluble contrast *via* an endoscope or catheter (water-soluble contrast challenge) may help exclude distal small-bowel obstruction (10, 11) (Figure 3).

Gastric motility may be impaired by peritoneal carcinomatosis (PC). One study reported markedly impaired gastric emptying in patients with gastric and pancreatic cancers after gastroduodenal stenting (12). Moreover, some underlying conditions, such as diabetes mellitus, are associated with severely impaired gastric motility. However, it is practically impossible to investigate the gastric emptying function prior to stent placement in patients with GOO.

### Factors associated with clinical failure

Eight previous studies have investigated the predictors of clinical failure for stenting in patients with GOO (Table 1). Clinical success is generally defined as the relief of obstructive symptoms and the improvement of oral intake. However, it should be noted that the definitions of clinical success differ among published studies. Although the most commonly used index to assess the level of oral intake is Gastric Outlet Obstruction Scoring System (GOOSS) proposed by Adler and Baron (20), Larssen et al. reported that the indicator was used only in 6 of the 41 published literatures (21). The definition reported in articles analyzing predictors of the outcome were shown in the annotation of Table 1. In addition, most reports did not specify when it was judged as clinical success.

**Table 1:** Predictors of clinical failure (success) of gastroduodenal stenting *via* multivariate logistic regression analyses.

**References**	**No. of cases**	**Etiology**	**Outcome analyzing association**	**Parameter**	**OR (95% CI)**	***P*** **-value**
Sasaki et al. (13)	97	All	Failure of solid food intake	KPS ≤ 50	3.65 (1.17–13.1)	0.03
				Ascites	3.2 (1.23–9.05)	0.02
Park et al. (14)	256 (including 39 cases with GJ)	Gastric cancer	Clinical success^a^	ECOG 3 or 4 (vs. ECOG 1)	0.1 (0.0–0.3)	Not shown
				Carcinomatosis with ascites (vs. no carcinomatosis)	0.3 (0.1–0.7)	Not shown
Sato et al. (15)	75	All	Poor effectiveness^b^	Peritoneal dissemination	9.94 (not shown)	0.01
Hori Y et al. (16)	126	All	Clinical failure^c^	KPS ≤ 40	1.19 (1.02–1.28)	0.041
				Peritoneal dissemination	1.20 (1.01–1.26)	0.038
				Stent expansion <30%^*^	1.55 (1.26–1.62)	<0.001
Yamao et al. (17)	278	All	Clinical ineffectiveness^d^	stenosis sites, no. ≥ 3	6.11 (2.16–17.3)	<0.01
				KPS ≤ 50	6.63 (2.89–15.2)	0.043
Shin et al. (18)	122	All	Clinical failure^e^	Gallbladder cancer	6.49 (1.51–59.66)	0.016
				Performance status (ECOG) ≥3	10.20 (2.44–42.72)	0.001
				Carcinomatosis (Yes)	35.71 (5.56–250.0)	<0.001
				Failure of endoscopic passage	6.95 (1.10–43.82)	0.039
Jang et al. (5)	183	All	Clinical success^f^	Moderate/severe vs. mildly/normal Malnutrition (albumin level)^**^	0.29 (0.13–0.65)	0.02
				Ascites	0.16 (0.07–0.36)	0.002
				Moderate/severe vs. none/mild ECOG scale	0.16 (0.05–0.48)	<0.001
Conti Bellocchi et al. (19)	112	All	Clinical success^g^	Age > 65	0.87 (0.73–0.98)	0.05
				D2 or D3	0.34 (0.19–0.84)	0.04
				Pancreatic	1.65 (1.19–4.39)	0.01

* *stent expansion rate on the procedure day*.

** *Nutrition level: normal nutrition: serum albumin ≥ 3.5 g/dL; mild malnutrition: albumin <3.5 g/dL, >3 g/dL; moderate malnutrition: albumin > 2.5 g/dL, <3 g/dL; severe malnutrition: albumin <2.5 g/dL*.

a *Defined as the patient's ability to tolerate oral intake without vomiting after either SEMS placement or palliative GJ*.

b *Defined as improvement of neither oral intake nor symptoms*.

c *Defined as no improvement of GOOSS score*.

d *Defines as the GOOSS scores of <2 and no relief from gastric outlet obstruction symptoms 7 days after stenting*.

e *Failure to see clinical success defined as improvement in the GOOSS scores 7 days after stenting*.

f *Defined as successful resumption of oral intake and relief of obstructive symptoms after either SEMS placement or GJ*.

g *Defined as the rate of patients experiencing at least 1 point in GOOSS score within 7 days from the procedure*.

*KPS, Karnofsky performance status; ECOG, ECOG performance status scale; GOOSS, Gastric Outlet Obstruction Scoring System*.

Of these eight studies, four studies indicated that PC was an independent predictor. The PC-related issues are described in detail in the next section.

Six of the eight studies showed that the performance scores [Karnofsky performance status (KPS) or Eastern Cooperative Oncology Group performance status (ECOG)] was a predictor of clinical failure (5, 13, 14, 16–18). Although various predictors associated with clinical failure of stenting in GOO, including PC and performance score, have been reported as shown in Table 1, a consensus regarding their significance has not been established. A study by Japanese researchers (13) showed that ascites and performance scores are predictors of solid food intake failure. There were reports that certain diseases, like gallbladder (18) and non-pancreatic cancer (19), were associated with clinical failure. Poor expansion on the day of the procedure (16), obstruction site (19), or malnutrition (5) were also predictive factors of clinical failure. Careful consideration should be given to alternative palliative methods in such cases prior to contemplating endoscpic stenting.

### Is PC a contraindication?

PC or dissemination could cause gastrointestinal dysmotility and the development of multiple bowel obstructions, and as a result, it is considered a relative contraindication to gastroduodenal stenting (6, 22). The guidelines established by the Cardiovascular and Interventional Radiological Society of Europe also indicate that PC is a relative contraindication for gastroduodenal stent placement (6).

In 2011, Mendelsohn et al. (23) reported that PC was not associated with clinical failure (23). However, the study did not investigate the predictors associated with clinical failure using multivariate analysis. A summary of carcinomatosis-related results extracted from the previous studies analyzing the predictors of clinical success using multivariate logistic regression shown in Table 2. The results regarding the association between clinical failure and the presence of PC are conflicting. Studies by Sasaki et al. (13) and Lee et al. (25) failed to show that PC was an independent predictor, similar to the results of Mendelsohns' study. Conversely, three studies, two from Japan (15, 16) and one from South Korea (18), concluded that PC was a predictor of clinical failure. Jeon et al. (24) demonstrated that PC with ascites was a predictor of clinical failure, but not PC alone. Despite including patients who underwent GJ in their study, Park et al. (14) encountered similar results depicting PC with ascites as an independent predictive factor of clinical failure (14). Pais-Cunha et al. (26) showed that PC is a predictor of early (postoperative day 7) and late (postoperative day 30) clinical failure. The authors also showed that the obstruction caused by PC was an independent predictor of clinical failure on days 7 and 30 (odds ratio (OR) [95% confidence interval (CI)], 9.7 [2.5–38.4], 7.6 [1.8–31.9], respectively) (26). Conversely, when PC was not the cause of obstruction, the association between PC and early and late clinical failure was rather weak (OR [95% CI], 2.8 [1.0–7.9], 1.77 [0.74–4.21]) (26).

**Table 2:** Carcinomatosis-related results extracted from studies analyzing predictors for clinical success *via* multivariate logistic regression analysis.

**References**	**No. of cases**	**Etiology**	**Outcome analyzing association**	**Parameter**	**OR (95%CI)**	***P*** **-value**
Sasaki et al. (13)	97	All	Failure of solid food intake	Peritoneal dissemination		
				No	1	
				Yes	1.83 (0.67–4.96)	0.24
Jeon et al. (24)	228	All	Clinical success	No carcinomatosis	1	
				Carcinomatosis without ascites	0.699 (0.209–2.330)	0.559
				Carcinomatosis with ascites	0.163 (0.058–0.461)	0.001
Park et al. (14)	256 (including 39 cases with GJ)	Gastric cancer	Clinical success	No carcinomatosis	1	
				Carcinomatosis without ascites	1.3 (0.4–5.2)	Not shown
				Carcinomatosis with ascites	0.3 (0.1–0.7)	Not shown
Lee et al. (25)	155	All	Clinical success	Peritoneal carcinomatosis		
				No	1	
				Yes	0.302 (0.050–1.829)	0.192
Sato et al. (15)	75	All	Clinical failure	Peritoneal dissemination		
				No	1	
				Yes	9.94 (not shown)	0.01
Hori et al. (16)	126	All	Clinical failure	Peritoneal dissemination		
				No	1	
				Yes	1.2 (1.02–1.26)	0.038
Shin et al. (18)	122	All	Clinical failure	Carcinomatosis		
				No	1	
				Yes	35.71 (5.556–250.000)	<0.001
Pais-Cunha et al. (26)	110	All	Worse early clinical outcome^†^	Carcinomatosis		
				No	1	
				Yes	4.8 (1.9–12.9)	<0.001
			Worse late clinical outcome^‡^	Carcinomatosis		
				No	1	
				Yes	4.3 (1.3–14.1)	0.008

† *Clinical success at 7 days*.

‡ *Clinical success at 30 days*.

However, the diagnosis of PC is not always easy. Peritoneal metastases were identified as nodular, plaque-like, or infiltrative soft tissue lesions in the peritoneal fat or peritoneal surface (27). CT plays a key role in PC diagnosis (27, 28). Magnetic resonance imaging with diffusion-weighted imaging and positron emission tomography/CT is sometimes superior to CT in identifying carcinomatous implants (28); however, there are some drawbacks, such as high cost and low accessibility. Ascites, parietal peritoneal thickening or enhancement, and small-bowel wall-thickening or distortion demonstrated positive predictive values of 72–93% (27). The diagnosis of PC is greatly influenced by nodule size. When the nodule diameter is 1 cm or greater, the detection rate of PC by multi-detector row CT is as high as approximately 90%, which is comparable to the surgical detection rate (29). However, when the nodule diameter is <1 cm, the detection rate is significantly reduced (29). In addition, small nodules of <5 mm in diameter could be detected on CT in only 11% of the cases (30). The detection rate varies depending on the region (30, 31). Koh et al. (30) reported that the detection of small-bowel involvement had much lower sensitivity compared with other regions (8 vs. 40–67%). Another study also showed similar results with poor sensitivity in the small bowel region (32), which is thought to have a significant impact on the effectiveness of the treatment. Furthermore, inter-observer differences in detection accuracy are likely to be significant (31).

The coexistence rates of PC in published studies have been highly divergent. Thus, the diagnostic accuracy of PC presumably differs between studies. Independent factors associated with clinical failure should be investigated after the correct diagnosis of PC. Therefore, a simplified and reliable test is necessary for the diagnosis of PC.

### Factors related to adverse events and stent dysfunction

Various adverse events (AEs) may occur after stent placement, some of which are critical. Even minor AEs require hospitalization and can significantly reduce a patient's quality of life. Surprisingly, the results of a recent review paper comprising pooled analysis from 2009 to 2015 (33) are comparable to those of a paper reviewing the data from 1998 to 2004 (34), with similar success and complication rates (Table 3). The latter systematic review article includes a lot of historical practices employed in the absence of a dedicated SEMS for GOO. From these results, it appears that the use of newer stents and more experience do not provide a significant benefit in improving outcomes. In a recent single-facility study by Reijm et al. comparing the results of two time periods (1998–2009 vs. 2010–2019) (35), the technical success rate was better in more recent years (1998–2009 vs. 2010–2019; 94 vs. 100%, *P* = 0.04). However, clinical outcome did not improve over time. A decreased GOO-symptom free survival and increased adverse event rate were noted in more recent years, which is probably due to an increased number of patients being treated with prior chemo- and/or radiation therapy in recent period (35). The authors also stated that another reason for the shorter symptom-free survival in stent-treated patients was that duodenal stent placement had been primarily recommended for patients with a shorter life expectancy, according to the results of the SUSTENT study in the early 2010s (36).

**Table 3:** Data related to the outcomes of gastroduodenal stenting by systematic reviews and pooled analyses.

**References**	**Publication year of reviewed articles**	**No. studies**	**No. cases**	**Technical success**	**Clinical success**	**RDO**	**Stent occlusion**	**Stent migration**	**Perforation**	**Bleeding**
Dormann et al. (33)	1998–2004	32	606	97.2% (589/606)	86.8% (526/606)	22.3%	17.2%	5.2%	0.7%	0.5%
van Halsema et al. (34)	2009–2015	19	1,281	97.3% (1,246/1,281)	85.7% (1,098/1,281)	19.6%	12.6%	4.3%	1.2%	4.1%

*RDO, recurrent duodenal obstruction*.

The predictors for AEs and stent dysfunction reported in the previous studies are shown in Tables 4, 5. The data shown in Table 4 are the results of studies using multivariate Cox regression analyses for predictor extraction, whereas those in Table 5 use multivariate logistic regression analyses. In a multicenter, retrospective study (42) that compared clinical outcomes and predictors of stent dysfunction between uncovered self-expandable metal stents (USEMS) and covered self-expandable metal stents (CSEMS), tumor ingrowth was found more frequently in the USEMS group, whereas stent migration occurred more often in the CSEMS group. Tumor ingrowth in USEMS was associated with a KPS score of >40, no presence of ascites, and insufficient (<30%) stent expansion on the day of the procedure (42). The authors presumed that patients with a good KPS and no ascites tended to have longer survival, leading to a greater likelihood of AEs. Meanwhile, stent migration in CSEMS was significantly associated with shorter stent length (*P* = 0.05) and post-stent chemotherapy (*P* = 0.03) (42). Another study (41) also indicated that the degree of stent expansion affects the outcome. It showed that a stent expansion rate of ≥75% on postoperative day 1 was an independent predictor of stent restenosis (41).

**Table 4:** Predictors of stent-related adverse events and stent dysfunction analyzed *via* multivariate Cox regression analyses.

**References**	**No. of cases**	**Etiology**	**Adverse events**	**Parameter**	**HR (95%CI)**	***P*** **-value**
Kim et al. (37)	213	All	Stent dysfunction (obstruction and migration)	Post-stent CT	0.19 (0.08–0.46)	<0.001
Cho et al. (38)	75	Gastric cancer	Stent dysfunction (obstruction)	CSEMS	0.29 (0.11–0.76)	0.01
				Post-stent CT	0.34 (0.13–0.91)	0.03
Kim et al. (39)	113	Gastric cancer	Stent migration	CSEMS	4.50 (1.52–14.33)	0.011
			Re-obstruction	Long TTP	0.29 (0.13–0.67)	0.004
				First-line CT	0.45 (0.22–0.93)	0.03
Miyabe et al. (40)	152	All	Stent dysfunction (any cause)	Post-stent CT	3.10 (1.14–9.00)	0.0264
Park et al. (14)	256 (including 39 cases with GJ)	Gastric cancer	Re-obstruction	ECOG 3 or 4 (vs. ECOG 1)	1.9 (1.1–3.1)	Not shown
				Previous chemotherapy	1.5 (1.1–2.0)	Not shown
				Carcinomatosis with ascites (vs. no carcinomatosis)	1.4 (1.0–2.0)	Not shown
Yamao et al. (17)	277	All	All AEs	CSEMS	0.27 (0.10–0.69)	<0.01
				Post-stent CT	0.42 (0.19–0.95)	0.04
			Stent dysfunction (any cause)	KPS ≤ 50	3.63 (1.55–8.50)	<0.01
			Stent migration	CSEMS	12.63 (2.35–67.80)	<0.01
			Perforation	Deployment of two stents	854.88 (11.36–64,356.6)	<0.01
Ye et al. (41)	87	All	Stent dysfunction (obstruction)	Stent expansion rate at day 1 ≥ 75%	0.12 (0.02–0.89)	0.04

*CT, chemotherapy; TTP, time-to-progression; CSEMS, covered self-expandable metal stent; KPS, Karnofsky performance status; ECOG, ECOG performance status scale*.

**Table 5:** Predictors of stent-related adverse events of stent dysfunction analyzed *via* multivariate logistic regression analyses.

**References**	**No. of cases**	**Etiology**	**Adverse events**	**Parameter**	**OR (95% CI)**	***P*** **-value**
Hori et al. (42)	126^*^	All	Tumor ingrowth in USEMS	KPS (>40%)	13.12 (1.16–148.18)	0.04
				Ascites (yes)	0.11 (0.02–0.66)	0.02
				Stent expansion rate at day 0 <30%	11.76 (2.35–58.89)	0.003
	126^**^	All	Stent migration in CSEMS	Stent length (<12 cm)	4.94 (0.98–25.02)	0.05
				Post-stent CT	5.01 (1.18–21.34)	0.03
Reijm et al. (35)	147	All	All AEs	Prior chemotherapy and/or radiotherapy	2.53 (1.17–5.47)	0.02
Kaneko et al. (43)	65	All	Biliary obstruction and/or pancreatitis	Female sex	9.16 (1.43–58.60)	0.02
				Absence of biliary stents	12.90 (1.84–90.20)	0.01
				Tumor invasion to the major papilla	25.80 (1.96–340.00)	0.01

* *Only for patients with USEMS*.

** *Only for patients with CSEMS*.

*AE, adverse event*.

As stated above, Reijms' study (35) showed that prior treatment with chemotherapy and/or radiotherapy was the only independent risk factor for AEs, and patients with a history of prior treatment experienced more AEs than those without (47 vs. 27%) (35). However, the reason for the discrepancy in AE incidence has not been alluded to in this study. The authors proposed that special attention be paid while informing patients who have received prior chemotherapy and/or radiotherapy to explain the benefits and potential risks of duodenal stent placement (35).

Yamao et al. reported that the deployment of two stents in the same session was involved in perforation and that CSEMS was associated with the development of stent migration (17). According to the authors, the overlapping of two stents may increase the axial force of the stents, leading to perforation (17). An article by Sasaki et al. on secondary gastroduodenal SEMS placed for revision (44) showed that gastrointestinal perforation occurred in four of 29 patients (13.8%). Stent used in these four patients were “WallFlex in WallFlex” in 3, “Niti-S in WallFlex” in one. The location of the perforation was assumed to be the contact site of the flare edge of WallFlex in all cases. Hence, the authors proposed that lower axial force SEMS should be chosen especially at the bending site either as a first stent or as a secondary stent (44). Although there is not enough evidence on this issue, using lower axial force SEMSs might help reduce the risk of perforation upon deployment of two SEMSs in long-segment stricture.

A recent Japanese study (43) reported that biliary obstruction or pancreatitis occurred in 18% of cases following duodenal stenting for D2 obstruction. The authors identified female sex, absence of biliary stents, and tumor invasion into the papilla as predictors of biliary obstruction and/or pancreatitis. The authors concluded that risk stratification can allow endoscopists to better identify patients at significant risk and permit detailed informed consent (43). Since biliary obstruction and pancreatitis can be caused by compression of the major papilla due to deployment of duodenal stents (45), the ampulla should not be covered as much as possible with a duodenal stent. However, as a systematic literature review assessing 19 studies patients (34) showed only two of 1,281 patients experienced pancreatitis, pancreatitis tend to generally be less common than other adverse events, presumably because most patients with D2 obstruction are pancreatic cancer with main pancreatic duct tumor involvement leading to pancreatic atrophy. It has been reported that pancreatitis after biliary SEMS placement less likely occur in patients with pancreatic cancer (46) and with main pancreatic duct tumor involvement (47), which may cause decreasing exocrine function. It is presumed that pancreatitis less likely occur due to such pathological conditions even after placement of duodenal stent covering the ampulla. Biliary obstruction also can develop by covering the ampulla with a duodenal SEMS. However, it appears manageable by EUS-guided or percutaneous biliary drainage without much difficulty, even in case of impossible transpapillary biliary drainage. Considering the invasiveness of GJ, ES is generally selected, even if it seems unavoidable to cover the ampulla with a SEMS.

### Impact of chemotherapy

The recent developments in intensive chemotherapy are expected to prolong survival in patients with gastric and pancreatic cancers and associated GOO. Chemotherapy after stent placement is helpful in extending the survival period in all carcinomas (40, 41, 48, 49), pancreatic cancers (50, 51) and non-pancreatic cancers (51).

A study by Miyabe et al., which used various stents, including the CSEMS (40), showed that post-stent chemotherapy increased the occurrence of stent migration, leading to poor stent patency (Table 4). Conversely, other studies successfully showed that chemotherapy after stent placement improved prolonged stent patency (37, 38) (Table 4). Stent migration would be more likely to occur in chemotherapy responders because responders would have a reduction in tumor size which could decrease the tension on a stent and allow it to move (39). On the other hand, responders are less likely to cause stent occlusion by causing tumor growth (39). Unlike other studies, Miyabe et al. (40) used CSEMS in as many as 68% of enrolled patients. Hence, it showed that chemotherapy was associated with poor stent patency (40), presumably because it seemed difficult to show preventive effect of chemotherapy against stent obstruction, while the increased risk of stent migration from chemotherapy seemed to be highly influenced.

The relationship between stent survival and the response to chemotherapy has also been reported. A study that dealt with gastric cancer (39) showed a lower rate of stent obstruction in patients with long time-to-progression (TTP) than in those with a short TTP (*p* < 0.001). Additionally, the administration of first-line chemotherapy (adjusted HR = 0.45, 95% CI = 0.22–0.93) was shown to be a protective factor against re-stenosis (39). A study on pancreatic cancer (52) showed a lower risk of stent dysfunction in responders than in non-responders among patients who received combination chemotherapy as the first-line treatment (*P* = 0.009) (52).

Chemotherapy after stent placement is safe and effective and thus should be considered for all patients with a reasonable physical, hemodynamic and functional status.

### Comparison with GJ

The focus in the early years was on superior short-term outcomes (shorter hospital stays, shorter time to diet), and thus stent placement was generally thought to be a better palliative procedure than GJ. A systematic review in 2007 showed that the ES-treated group had less frequent recurrent obstructive symptoms than the GJ-treated group (53). This result was also demonstrated in the SUSTENT study, the largest randomized comparative trial (RCT) to date, comparing ES with GJ (36). Since then, an increasing number of reports suggest that GJ may be preferable in the long term. Recent meta-analyses comparing ES with GJ showed that ES is likely to have a higher possibility of recurrent obstruction, necessitating re-intervention, although they have favorable short-term outcomes, such as shorter hospital stay and time to diet (54, 55). Therefore, the GOO guidelines from the American Society for Gastrointestinal Endoscopy (ASGE) (56) and Clinical Practice Update from the American Gastroenterological Association (AGA) (7) proposed that GJ be considered for patients with longer life expectancy, good functional status, and surgical fit. However, ES should be considered if patients are not eligible for GJ (7, 56). The SUSTENT study proposed that ES is preferable for patients with a life expectancy of <2 months. Their proposal was based on findings that at the 2 month follow-up, the surgical procedure was more effective than stent placement (36). However, the ASGE panel agreed to set the cut-off for treatment decisions to 6 months while creating the recommendations (56).

In accordance with the SUSTENT study (36) and the ASGE (56) and AGA (7) guidelines, suitable candidates for ES should be patients with short-life expectancy and poor functional status. Although many researchers have reported chemotherapy prolongs the patency of gastroduodenal stent, such patients are often not indicated for chemotherapy. According to a study on ES over the past 20 years by Reijm et al. (35), only 12% of patients underwent concurrent chemotherapy, although 33% received prior chemotherapy and/or radiotherapy. However, there are some cases in which PS improves after stent placement by alleviating obstructive symptoms and resuming oral intake. If the patient is considered to tolerate chemotherapy due to the improvement in functional status after stent placement, chemotherapy should be introduced as much as possible.

Nonetheless, predicting life expectancy is not always easy, whether the borderline period is 2 or 6 months. The results of previous studies regarding the predictors of survival in patients with malignant GOO are shown in Table 6. Various predictors have been reported (40, 41, 48, 49, 57–59). Further studies are warranted to establish suitable predictors of survival time.

**Table 6:** Predictors for survival of patients with GOO assessed *via* multivariate Cox regression analyses.

**References**	**No. cases**	**Etiology**	**Treatment procedure**	**Parameter**	**HR (95%CI)**	***P*** **-value**
van Hooft et al. (57)	105	All	ES	Pain medication (other morphines)	2.42 (1.38–4.25)	0.002
				WHO-PS (0–2 vs. 3–4)	2.63 (1.68–4.12)	<0.001
				QLQ-C30 (pain)	1.01 (1.00–1.01)	0.035
Jeurnink et al. (58)	151	All	ES or GJ	WHO-PS (0–1 vs. 2–4)	2.2 (1.69–2.88)	<0.001
Ye et al. (48)	71	All	ES	Tumor origin (gastric)	0.25 (0.06–0.97)	0.045
				Carcinomatosis (yes)	3.09 (1.04–9.19)	0.04
				Post-stent CT	0.38 (0.19–0.76)	0.006
Miyabe et al. (40)	152	All	ES	Post-stent CT	0.60 (0.39–0.90)	0.0132
				Stage IV or post-op cancer recurrence	1.75 (1.07–3.00)	0.0252
				Attainment of hospital discharge	0.26 (0.16–0.44)	<0.0001
				KPS ≥ 60%	0.58 (0.38–0.91)	0.0174
Oh et al. (51)	196	Panc ca	ES	Post-stent CT	0.35 (0.25–0.48)	Not shown
				Absence of distant metastasis	0.64 (0.48–0.87)	Not shown
	96	Nonpanc ca	ES	Post-stent CT	0.40 (0.23–0.70)	Not shown
				Absence of distant metastasis	0.48 (0.28–0.83)	Not shown
Kobayashi et al. (50)	71	Panc ca	ES	UICC stage (IV vs. II/III)	3.73 (1.72–8.10)	<0.001
				NLR (≥5)	2.69 (1.47–4.91)	<0.001
				Post-stent CT (no)	1.85 (1.02–3.38)	0.045
Ye et al. (41)	87	All	ES	Tumor origin (non-gastric)	2.41 (1.40–4.17)	0.002
				Carcinomatosis (yes)	2.54 (1.43–4.51)	0.001
				Post-stent CT	0.55 (0.32–0.94)	0.03
Sugiura et al. (59)	129	Panc ca	ES or GJ	Liver metastasis (presence)	1.90 (1.27–2.87)	0.002
				NLR (≥4)	4.01 (2.54–6.34)	<0.001
				Cancer pain (presence)	2.08 (1.40–3.09)	<0.001
Wei TH et al. (49)	79	All	ES	Length of stenosis (≥4 vs. <4 cm)	1.92 (1.06–3.49)	0.032
				Post-stent CT	0.33 (0.17–0.63)	0.001

*ES, endoscopic stenting; GJ, gastrojejunostomy; Panc ca, Pancreatic cancer; Nonpanc ca, Non-pancreatic cancer; NLR, neutrophil-to-lymphocyte ratio; CT, chemotherapy; QLQ-C30, European Organization for the Research and Treatment of Cancer Quality of Life Questionnaire*.

In addition, three comparable studies between ES and GJ using propensity score matching analysis have shown that GJ is better than ES in reducing long-term AEs and improving patient survival (5, 60, 61). Out of the three studies, the one (60) that dealt with gastric cancer showed GJ was associated with lesser frequency of reintervention (5 vs. 29%, *P* = 0.003), better nutrition status after the procedure (serum albumin change: +0.75 g/dl vs. −0.15 g/dl, *P* = 0.002) and longer chemotherapy tolerance duration (median tolerance without dose reduction: 243 vs. 74 days, *P* = 0.006) (60). These effects may presumably result in more favorable patient survival. The most recent meta-analysis (4) reported that GJ had a better survival rate than ES in the gastric cancer group (HR, 0.33; *P* = 0.009). However, no statistically significant difference was observed in the pancreatic cancer group (HR, 0.55; *P* = 0.159).

Endoscopic ultrasound-guided gastrojejunostomy (EUS-GJ) has emerged as an alternative to surgical GJ (SGJ) and stent placement. A meta-analysis that compared EUS-GJ with enteral stenting reported comparable success rates and lower rates of reintervention in EUS-GJ (4 vs. 23.6%, *P* = 0.001) (62). A meta-analysis that compared EUS-GJ with SGJ showed that EUS-GJ was superior in terms of clinical success, lower overall AE, shorter procedure time, and shorter post-procedure hospital stay (63). Although there are no officially approved dedicated devices for EUS-GJ in some countries, and the procedure requires experienced hands, it will be necessary to create a treatment decision algorithm that also includes EUS-GJ based on the patient's condition in the future.

## What kind of stent is preferable?

### Stent structure

Currently, various dedicated SEMSs for GOO from multiple manufacturers are commercially available. Most of the available SEMSs are braided with wires made of an alloy of nickel and titanium (Nitinol). There have been four reports comparing the clinical outcomes of two different USEMSs, despite studies with a limited number of cases (48, 64–66) (Table 7).

**Table 7:** Difference in clinical outcomes between two different uncovered SEMS.

**References**	**Study design**	**Stent (structure)**	**No cases**	**Tech success**	**Clin success**	**AEs**	**RDO**	**Reintervention**
Maetani et al. (64)	Retro	Ultraflex (knitted) vs. Niti-S (braided, hook)	31 vs. 53	100 vs. 98.1%	28 (90.3%) vs. 50 (94.3%)	3 (9.7%) vs. 3 (5.7%)	2 (6.5%) vs. 11 (20.8%)	1 (3.2%) vs. 11 (20.8%) *P* = 0.049
Kato et al. (65)	Retro	WallFlex (braided, cross) vs. Niti-S (braided, hook)	46 vs. 79	100 vs. 100%	84.8 vs. 96.2% *P* = 0.023	5 (10.9%) vs. 10 (12.7%)	8 (17.4%) vs. 13 (16.5%)	Not shown
Okuwaki et al. (66)	RCT	WallFlex (braided, cross) vs. Niti-S (braided, hook)	14 vs. 17	100 vs. 100%	93 vs. 88%	4 (29%) vs. 4 (24%)	9 (64%) vs. 4 (24%) *P* = 0.027	Not shown
Ye et al. (48)	Retro	WallFlex (braided, cross) vs. Bonastent (braided, hook and cross)	41 vs. 30	100 vs. 100%	Not shown	17 (41.5%) vs. 9 (30%)	14 (34.1%) vs. 8 (26.7%)	10 (24.4%) vs. 4 (13.3%)

*Retro, retrospective cohort study; RCT, randomized controlled trial; hook, hook wire type structure; cross, cross wire type structure; hook and cross, hook and cross wire type structure*.

**Figure 4:**
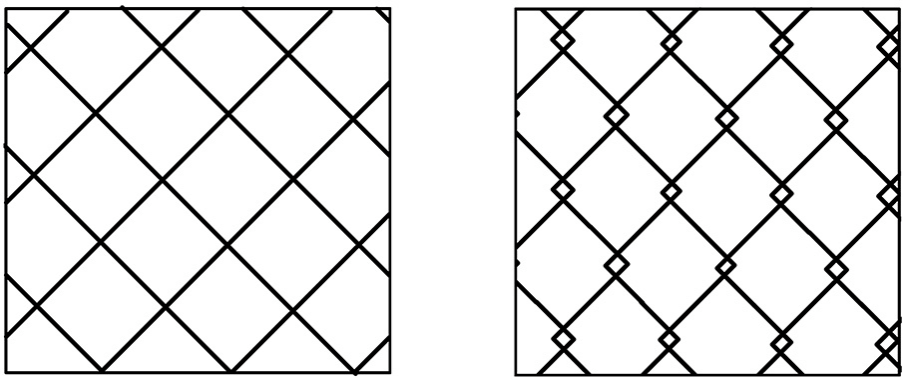
Two types of braided SEMS. (Left) cross wire type, (Right) hook wire type.

A study that compared a knitted stent (Ultraflex) with a braided stent (Niti-S) (64) showed a higher rate of recurrent duodenal obstruction (RDO) and a higher reintervention rate in patients treated with braided Niti-S stents. This result was presumed to be due to the different etiologies between the two groups; Ultraflex-treated patients tended to die before the onset of RDO due to the shorter survival period of the group (64). Three studies compared the different braided wire structures. Braided SEMS can be classified into two structures: cross wires and hook wires (Figure 4). Hook-wired SEMS have unfixed cells with a weaving construction, which contributes to their marked flexibility. An experimental study using various SEMS, despite an experiment using colorectal stents, reported that most hook-wired SEMS were extremely flexible, which is expected to reduce the pressure load on the intestinal wall for clinical use, thereby decreasing the risk of adverse events (67).

Two studies from Japan comparing a braided, cross-wired SEMS (WallFlex) and a braided, hook-wired SEMS (Niti-S) (65, 66) showed that Niti-S was associated with favorable results. A retrospective study by Kato et al. reported higher clinical success in patients with Niti-S than in those with WallFlex, although the background characteristics of the two groups were not noted. The authors stated that the high flexibility of Niti-S appears to be suitable for angulated anatomy (65). A randomized comparison by Okuwaki et al. (66) reported more frequent RDOs in patients who received WallFlex (64 vs. 24%; *P* = 0.027), primarily because of kinking, despite no difference in clinical success. The authors assumed that the higher axial force of WallFlex did not allow the stent to accommodate angulation in the duodenum (66). A study by Taiwanese researchers compared cross-wired SEMS (WallFlex) and hook and cross-wired SEMS (Bonastent) and failed to show a difference in the clinical outcome.

Studies with each stent in a separate prospective cohort have been reported by the same Dutch researchers, though it was not a comparative study (68, 69). The clinical success rate and AE rates of WallFlex and Niti-S were 84 vs. 77% and 27 vs. 35%, respectively, thus showing a somewhat better result for WallFlex (68, 69). A large-scale prospective study of >200 cases using WallFlex showed satisfactory clinical outcomes, with a clinical success rate of 91% and an AEs rate of 20.3%, including RDOs (70). A retrospective study conducted by Indian researchers, which dealt with 214 cases using WallFlex (71), also reported favorable results, with rates of achieving clinical success in 91%, AEs in 11%, and RDOs in 31%. No fatal complications, including perforations, were observed. However, because the stent placement strategies (i.e., selection of stent length and deployment configuration) may have differed among the studies, it is impossible to determine a suitable stent structure for GOO.

### CSEMS

**Figure 5:**
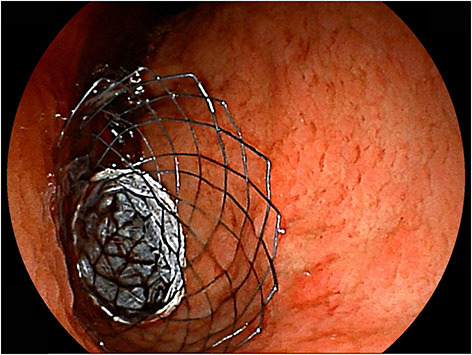
Placement of covered SEMS. A patient with antral gastric cancer was treated with partially-covered SEMS.

The CSEMS was developed with the expectation of reducing the risk of stent blockage due to tumor ingrowth and hyperplasia (Figure 5). Stent wires are not embedded into the gastrointestinal wall because of the presence of a covering membrane, which may lead to a higher risk of stent migration. Although a recent meta-analysis from seven randomized controlled trials and nine observational studies (72) also showed CSEMS was associated with a higher rate of migration, CSEMS eventually performed better with prolonged stent survival compared with uncovered SEMS (USEMS) (HR:0.68, 95% CI: 0.48–0.96, *P* = 0.03) (72). However, a subsequently published large-scale RCT comparing CSEMS and USEMS (73) showed conflicting results, with better overall stent patency in USEMS (35.2 vs. 23.4%, *P* = 0.01). Nevertheless, the risk of stent migration remains high with CSEMS, and stent designs with various anti-migration properties have been developed and evaluated. An RCT comparing CSEMS and USEMS in patients with gastric cancer (74) was conducted. The CSEMS used in this study was designed to have a reduced radial force and indentation in the central part of the SEMS, with an uncovered flared portion at both ends. Despite no statistically significant difference in migration rate between both groups, the migration rate was rather high at 9.8% in CSEMS (74). A study with the use of partially-covered “big cup” SEMS was prematurely terminated because proximal migration occurred in three out of six patients (75). Choi et al. evaluated patients with GOO who were treated with a newly designed, partially covered SEMS that had star-shaped wing flaps at the proximal end to reduce distal stent migration (76). In this study, proximal migration occurred in 11.1%, with no distal migration (76). Therefore, there is a need to develop more efficient antimigration systems.

Some fixation techniques have been described for CSEMS. A pilot study that assessed the efficacy of over-the-scope clips for gastroduodenal stent fixation reported stent migration in only one case (6.7%) (77). Currently, over-the-scope clips dedicated to stent fixation (OTSC^®^ STENTFIX, Ovesco Endoscopy AG, Tübingen, Germany) are commercially available (78). Despite studies dealing with primarily benign diseases, endoscopic suturing was shown to be helpful in mitigating the risk of stent migration (79). These fixation devices may complement the antimigration system of the CSEMS.

## Choice of biliary intervention in cases with coexisting biliary obstruction

Patients with periampullary cancer frequently experience biliary and duodenal obstructions. In such conditions, intervention is required for both the biliary tract and duodenum. The double-stenting procedure is a widely used, less invasive, and rapidly effective alternative to conventional double-bypass surgery. A recent meta-analysis (80) established favorable results, with a high success rate and less frequent AEs in the double stenting procedure but a more frequent need for reintervention (21% [16–27%] vs. 10% [4–19%]) (80).

**Figure 6:**
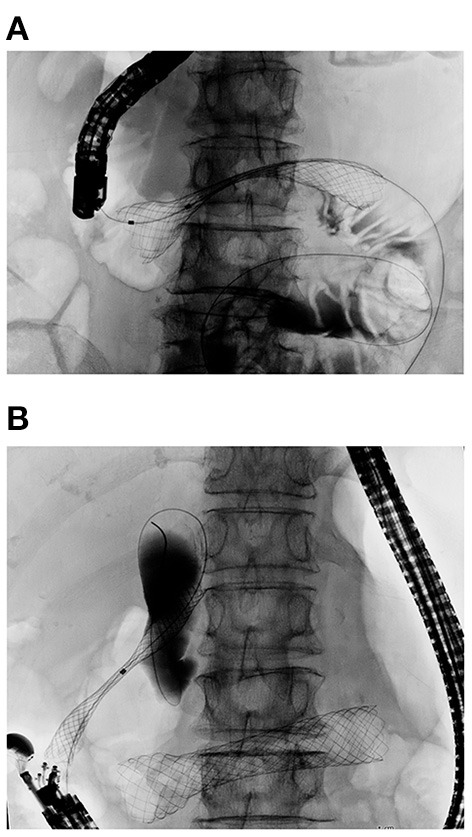
Double stenting procedure for concurrent biliary and duodenal obstruction. **(A)** Duodenal stent placed first for D3 obstruction. **(B)** EUS-CDS was carried out after full expansion of the duodenal stent to avoid duodenobiliary reflux. A SEMS was inserted into the common bile duct and deployed in the proper position.

There are three types of nonsurgical biliary interventions: (i) endoscopic transpapillary biliary drainage (ETBD) by endoscopic retrograde cholangiopancreatography, (ii) percutaneous biliary drainage, and (iii) endoscopic ultrasound-guided biliary drainage (EUS-BD) (Figure 6). A meta-analysis (80) also proposed that ETBD can be recommended as a first-line treatment for cases with coexisting biliary obstruction because of its lower AE rate compared to percutaneous biliary drainage or EUS-BD (80).

Combined biliary and duodenal obstructions were classified by Mutignani et al. according to the site (Type I–III) and sequence (Groups 1–3) (81). The condition in which both obstructions occur concurrently (group 2) is likely to be difficult to manage. In such cases, endoscopic retrograde cholangiopancreatography often requires prior balloon dilation for duodenal stenosis if ETBD precedes duodenal stenting (81). Conversely, if a duodenal stent is placed first, the papilla should not be covered with a duodenal stent as much as possible to allow transpapillary biliary access. In patients with preexisting duodenal stents that cover the papilla, ETBD is quite difficult, even by expert endoscopists, with a success rate of 22.2–31.6% (82, 83). In cases of failure, percutaneous biliary drainage or EUS-BD must be performed as a salvage procedure.

Recently, EUS-BD has rapidly become popular, as there is no need to wait for the fistula maturation of an uncomfortably placed percutaneous catheter. A study that compared clinical outcomes of EUS-BD and ETBD in patients with indwelling duodenal stents, the rate of stent dysfunction tended to occur less frequently in EUS-BD than ETBD (14 vs. 54%; *P* = 0.157) despite similar AE rates (84). A comparative study between EUS-hepaticogastrostomy (EUS-HGS) and EUS-choledochoduodenostomy (EUS-CDS) for patients with combined biliary and duodenal obstructions reported favorable outcomes with EUS-HGS because of longer stent patency and less frequent AEs (85). Hamada et al. (86) reported that the presence of a duodenal stent might deteriorate biliary stent patency, presumably because of duodenobiliary reflux (DBR). A study investigating 109 patients who underwent double stenting (87) showed that CSEMS, as a duodenal stent, helped prolong biliary stent patency. The reason for this is presumed to be the DBR prevention effect of the duodenal CSEMS, although the number of cases in which the ampulla was covered has not been reported (87).

An international, multicenter study revealed that the time to recurrent biliary obstruction (TRBO) did not differ significantly by timing (Group 1 vs. Group 2 vs. Group 3) or location (Type I vs. Type II vs. Type III) of duodenal obstruction (*P* = 0.30 and 0.79, respectively) (88). Conversely, some studies have indicated that positional relationship between the biliary and duodenal stents may influence the outcome. A retrospective study assessing patients who underwent double stenting showed that duodenal stent dysfunction and the biliary stent end located above the duodenal stent were risk factors for biliary stent dysfunction (89). A similar study by Taiwanese researchers reported that duodenal obstruction below the papilla and a score of ≤ 2 on the GOOSS (20) after treatment for duodenal obstruction were associated with DBR-related biliary CSEMS dysfunction (90). More attention should be paid to formulating a treatment strategy for patients with combined biliary and duodenal obstruction, considering the possible risk of developing DBR.

Chemotherapy after double stenting may prolong survival. According to a study that investigated pancreatic cancer patients with double stenting, multivariate analysis identified chemotherapy post double stenting (OR: 0.19; 95% CI: 0.059–0.60; *P* = 0.0051), reintervention for biliary stent dysfunction (OR: 0.21; 95% CI, 0.081–0.50; *P* = 0.0002), and performance status (<2) (OR: 0.28; 95% CI: 0.098–0.71; *P* = 0.0064), are independent predictors of patient survival. When assessing only patients with PS < 2, the median survival time was significantly longer in patients who received chemotherapy after double stenting than in those who did not (175 vs. 77 days, *P* = 0.0029) (91). Hence, post-stent chemotherapy may be considered, even in patients with both biliary and duodenal stenting.

## Conclusion

- Stent placement may cause functional failure in ~10% of cases, which can be reduced by sufficient preparation.
- A thorough radiological examination should be considered before the procedure to exclude absolute contraindicated cases.
- Careful consideration is required before making treatment decisions for patients with PC and ascites or poor performance scores.
- GJ is recommended for patients with a life expectancy of >2 months and a good functional status. But it should be kept in mind that estimating life expectancy may not always be easy because there is no absolute guaranteed predictor for survival at the moment.
- SEMS with a hook wire structure seems suitable, particularly in angulated anatomical areas, owing to the lower axial force. However, there is currently no consensus on the ideal stent structure.
- The CSEMS has a trade-off between the blockage prevention effect and being prone to migration. The development of effective anti-migration systems for CSEMS is required.
- In cases with combined biliary and duodenal obstruction, attention should be paid to the risk of DBR for ETBD according to the location of duodenal obstruction.

## Author contributions

IM participated in all the work of this article, including participation in the concept, design, analysis, writing, and revision of the manuscript.

## Conflict of interest

The author declares that the research was conducted in the absence of any commercial or financial relationships that could be construed as a potential conflict of interest.

## Publisher's note

All claims expressed in this article are solely those of the authors and do not necessarily represent those of their affiliated organizations, or those of the publisher, the editors and the reviewers. Any product that may be evaluated in this article, or claim that may be made by its manufacturer, is not guaranteed or endorsed by the publisher.
